# Predictors of localization, outcome, and etiology of spontaneous intracerebral hemorrhages: focus on cerebral amyloid angiopathy

**DOI:** 10.1007/s00702-020-02174-2

**Published:** 2020-03-19

**Authors:** Bernadett Fakan, Zita Reisz, Denes Zadori, Laszlo Vecsei, Peter Klivenyi, Levente Szalardy

**Affiliations:** 1 Department of Neurology, Faculty of Medicine, Albert Szent-Györgyi Clinical Center, University of Szeged, Semmelweis u. 6, 6725 Szeged, Hungary; 2 Department of Pathology, Faculty of Medicine, Albert Szent-Györgyi Clinical Center, University of Szeged, Állomás u. 2, 6725 Szeged, Hungary; 3 MTA-SZTE Neuroscience Research Group, Semmelweis u. 6, 6725 Szeged, Hungary

**Keywords:** Cerebral amyloid angiopathy, Epidemiology, Intracerebral hemorrhage, Lobar, Predictor

## Abstract

Despite its clinical relevance, cerebral amyloid angiopathy (CAA) is underdiagnosed worldwide. This retrospective study aimed to assess the incidence, etiology, predictors, and outcome of intracerebral hemorrhages (ICHs) in this region, with special focus on possible underlying CAA. Database screening of acute cares with intracranial hemorrhage diagnosis within 01/07/2014–01/07/2018 were conducted analyzing medical records and imaging. Spontaneous ICHs were classified as deep (basal ganglionic/thalamic/brainstem) and lobar/cerebellar (i.e., CAA-compatible) ICHs. Probable/definite CAA was established using the modified Boston criteria in a subgroup with ‘complete’ radiological/neuropathological work-up. The ability of several factors to discriminate between deep and lobar/cerebellar ICHs, between probable/definite CAA and non-probable CAA cases, and to predict 1-month case fatality was assessed. Of the 213 ICHs identified, 121 were in deep and 92 in lobar/cerebellar localization. Sub-analysis of 47 lobar/cerebellar ICHs with ‘complete’ work-up identified 16 probable/definite CAA patients, yielding an estimated 14.7% prevalence of CAA-related ICHs. Chronic hypertension was the most prevalent risk factor for all types of ICHs (including CAA-related), with hypertensive excess and younger age being independent predictors of deep whereas antiplatelet use of lobar/cerebellar localization. The 1-month case fatality was 33.8%, driven predominantly by age and INR > 1.4. Probable/definite CAA diagnosis was independently predicted by age, prior intracranial hemorrhage, and antiplatelet use. First in this region and among the few in the literature, this study reports a remarkable prevalence of CAA-related ICHs, emphasizing the need for an increased awareness of CAA and its therapeutic implications, especially regarding antiplatelets among the elderly.

**Electronic supplementary material:**

The online version of this article (10.1007/s00702-020-02174-2) contains supplementary material, which is available to authorized users.

## Introduction

Stroke is one of the leading causes of death and disability globally. After ischemic stroke, intracerebral hemorrhage (ICH) is the second most prevalent type, accounting for some 10% of all cases (Feigin et al. 2009). The most common known risk factors of spontaneous ICHs include advanced age, chronic hypertension, cerebral amyloid angiopathy (CAA), alcohol and drug abuse, and antithrombotic medications (Aguilar and Brott 2011). Recent studies further highlight the role of systemic inflammation in determining ICH propagation and outcome, and implicate the potential protective role of substances with anti-inflammatory properties (Di Napoli et al. 2014, 2016).

Based on etiological considerations, ICHs are commonly classified into deep ICHs (i.e., originating from blood vessels in the basal ganglia, thalamus, or brainstem), predominantly associated with chronic hypertension/hypertensive arteriopathy, and lobar ICHs, which are most frequently related to CAA and, relatively less frequently, to other etiologies such as vascular malformations or tumors (Ikram et al. 2012). The distribution of cerebral microbleeds (CMBs) follows a similar anatomical pattern, with chronic hypertensive (a.k.a. hyaline) arteriopathy associating primarily with CMBs in deep localizations and CAA typically associating with lobar CMBs, sparing the deep structures (Greenberg et al. 2009). Of note, cerebellar ICHs and CMBs can be attributable to either chronic hypertensive arteriopathy or CAA (Greenberg and Charidimou 2018); therefore, their classification in the literature is not unequivocal, some authors classifying them as deep (Labovitz et al. 2005) or non-lobar (Zia et al. 2007; Samarasekera et al. 2015) while others omitting them from analyses (Falcone et al. 2013), analyzing them separately (Samarasekera et al. 2012), or using alternative classifications (Gregoire et al. 2010).

In CAA, cortical and leptomeningeal small arteries/arterioles and capillaries are degenerated due to the progressive deposition of β-amyloid peptides, associating with both CAA-related ischemic alterations (including microinfarctions and leukoaraiosis) and different types of hemorrhages [including CMBs and ICHs in lobar (and cerebellar) localization, and cortical superficial siderosis (CSS, a.k.a. convexity subarachnoid hemmorhage (SAH))] (Yamada 2015).

Clinical manifestations of CAA can be various, ranging from asymptomatic stage to fatal ICHs. Typically, CAA-related ICHs are recurrent and cause various neurological deficits (depending on localization), headache, and epileptic seizures with or without loss of consciousness (Yamada 2015). Patients usually develop slowly progressive cognitive impairment due to multiple CMBs and microinfarctions (i.e., vascular neurocognitive disorder or mixed-type neurocognitive disorder, in the presence of concomitant Alzheimer’s disease (AD) pathology). In addition, CAA patients frequently experience transient ischemic attack (TIA)-like events, a.k.a. transient focal neurological episodes (TFNEs) or ‘amlyoid spells’; these events are, however, presumed to be due to focal epileptic activity secondary to CSS and not thrombotic in origin (Yamada 2015).

Magnetic resonance imaging (MRI) sequences sensitive to susceptibility artifacts generated by hemosiderin deposits of previous micro- and macrobleeds, such as gradient echo (GRE), susceptibility-weighted imaging (SWI), or T2* sequences, in addition to a set of clinical characteristics, make it possible to establish a probabilistic diagnosis of CAA in vivo with high diagnostic accuracy (Greenberg and Charidimou 2018). The original Boston criteria was modified in 2010 recognizing CSS as part of CAA-related alterations (Linn et al. 2010). This Modified Boston criteria (with increased sensitivity and retained specificity) enables establishing the diagnosis of possible CAA and probable CAA without histological confirmation (i.e., biopsy specimen or post mortem tissue) in patients above 55 years, with the presence of a single hemorrhagic alteration (ICH, CMB, or CSS) in lobar localization without other cause allowing the diagnosis of possible CAA, and > 1 of such hemorrhagic alterations without other cause meeting the diagnosis of probable CAA (Linn et al. 2010). While deep (basal ganglionic, thalamic, and brainstem) hemorrhagic alterations preclude the diagnosis of both possible and probable CAA, cerebellar bleeds are allowed, albeit not counted for the diagnosis (Greenberg and Charidimou 2018). Definite CAA diagnosis can be established only via full post mortem histological investigation.

CAA is by no means infrequent. Affecting some 5% of the population over 65 years of age (Biffi and Greenberg 2011), its prevalence is comparable to that of AD (Lobo et al. 2000) [with which it shows some 70–80% overlap (Jellinger 2002; Brenowitz et al. 2015)] and atrial fibrillation (AF) (Majeed et al. 2001). Though curative therapy is lacking, the clinical relevance of the diagnosis is high. Indeed, the use of anticoagulants is contraindicated in CAA according to current guidelines due to a 7–tenfold increase in the risk of ICH (Kernan et al. 2014; Heidbuchel et al. 2015). The use of antiplatelet therapy should also be carefully considered in CAA, due to an up to fourfold increase in the risk of recurrent ICH in general population after lobar ICH (Biffi et al. 2010) and a twofold prevalence of lobar CMBs in patients suffering ICH while on antiplatelet therapy (Gregoire et al. 2010). Though prior ICH has always been an absolute contraindication for systemic thrombolysis in acute ischemic stroke, a > 10 CMB number per se has recently been introduced as a contraindication due to uncertain benefit (Powers et al. 2018). Despite the high prevalence and relevance among the elderly, the establishment of a clinical diagnosis of CAA to our experience has been sporadic. Based on the discrepancy between the expected frequency of CAA among the elderly and the experienced occurrence of CAA diagnosis in the routine clinical practice, our aim was to asses the frequency of the different types of spontaneous ICHs observed in our stroke center with special focus on estimating the underlying prevalence of CAA, by means of the retrospective re-evaluation of written and imaging documentation. Emphasis was given on the analysis of the predictive value of putative risk factors for ICH location, probable/definite CAA diagnosis, and fatal outcome.

## Materials, patients, and methods

Via screening the electronic database, patients who received acute in-patient care in our center between 01/07/2014 and 01/07/2018 with any of the intracranial hemorrhage-related International Classification of Diseases (ICD) diagnosis codes were identified. Reviewing the imaging scans and medical records, spontaneous ICHs were separated from intracranial hemorrhages with traumatic etiology, cases with SAH, primary intraventricular hemorrhage, and hemorrhagic transformation of ischemic stroke, and from cases with inadequate coding. Spontaneous ICHs were further classified according to hematoma localization as deep ICHs (basal ganglia, thalamus, or brainstem) and lobar/cerebellar ICHs (regions compatible with the diagnosis of probable CAA, enabling the estimation of the prevelance of underlying CAA).

The prevalence of different etiologies behind lobar/cerebellar ICHs was assessed in a subpopulation who underwent ‘complete’ clinical work-up, defined as being subjected to computed tomography angiography (CTA) or magnetic resonance angiography (MRA) as well as MRI-SWI (if structural etiology was not identified by angiography) and/or post mortem neuropathological work-up. Definite CAA, probable CAA, and possible CAA diagnoses were retrospectively established or revised as per the Modified Boston criteria (Linn et al. 2010). An ICH was considered CAA-related if it met the criteria for probable and/or definite CAA.

Clinical data collected about the patients included their age at the time of ICH, sex, history of intracranial vascular events (including TIA, clinical episode of ischemic or hemorrhagic stroke), family history of any stroke, prior episodes of loss of consciousness, chronic hypertension, hypertensive excess at presentation (defined as ≥ 180 mmHg systolic blood pressure), current use of antiplatelet and/or anticoagulant drugs, as well as the international normalized ratio (INR) values on admission for ICH. Case fatality (lethality) was defined as fatal outcome within 1 month secondary to the ICH event in the absence of evidence for unrelated cause of death (e.g., cardiac arrest).

For statistical analysis, the SPSS 20.0 software (IBM Corp., Armonk, New York, USA; RRID:SCR_002865) was used. For comparative assessment of continuous variables, parametric (Student *t*) or non-parametric (Mann–Whitney *U*) tests were used after normality analysis with the Shapiro–Wilk test. For comparative analysis of discrete variables, cross-tabulation analysis was used by the Chi^2^ test, applying Fisher’s exact values when appropriate. Backward conditional multivariate binary logistic regression analyses were used to assess the effect of predictors found to be significant in the univariate comparative analyses. The binary outcomes were deep ICHs vs. lobar/cerebellar ICHs, case fatality at 1 month vs. alive at 1 month, and probable/definite CAA vs. non-probable CAA (including all deep ICHs and the subgroup of lobar/cerebellar ICH with ‘complete’ clinical work-up not meeting the criteria for probable/definite CAA). The level of significance was *p* < 0.05. Data within the text are presented as mean ± standard error of the mean (SEM) or median [interquartile range] in the cases of normal or non-normal distribution, respectively.

## Results

### Revision of diagnoses, estimation of CAA-related ICH prevalence

**Fig. 1:**
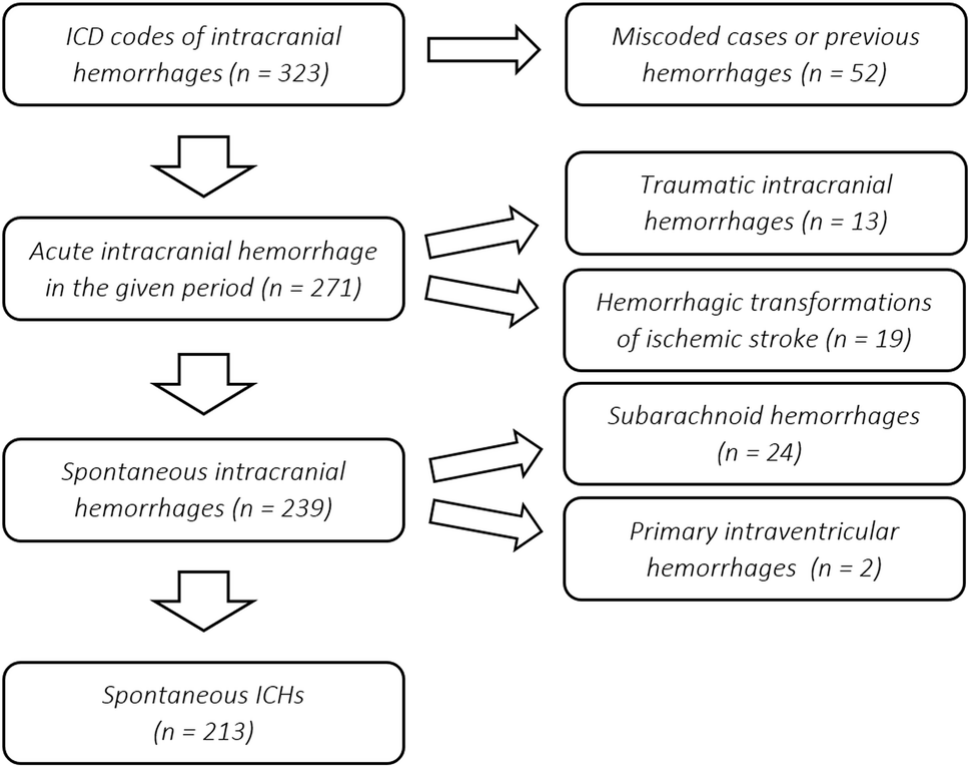
Flow diagram of the process of identifying spontaneous intracerebral hemorrhages (ICHs)

**Fig. 2:**
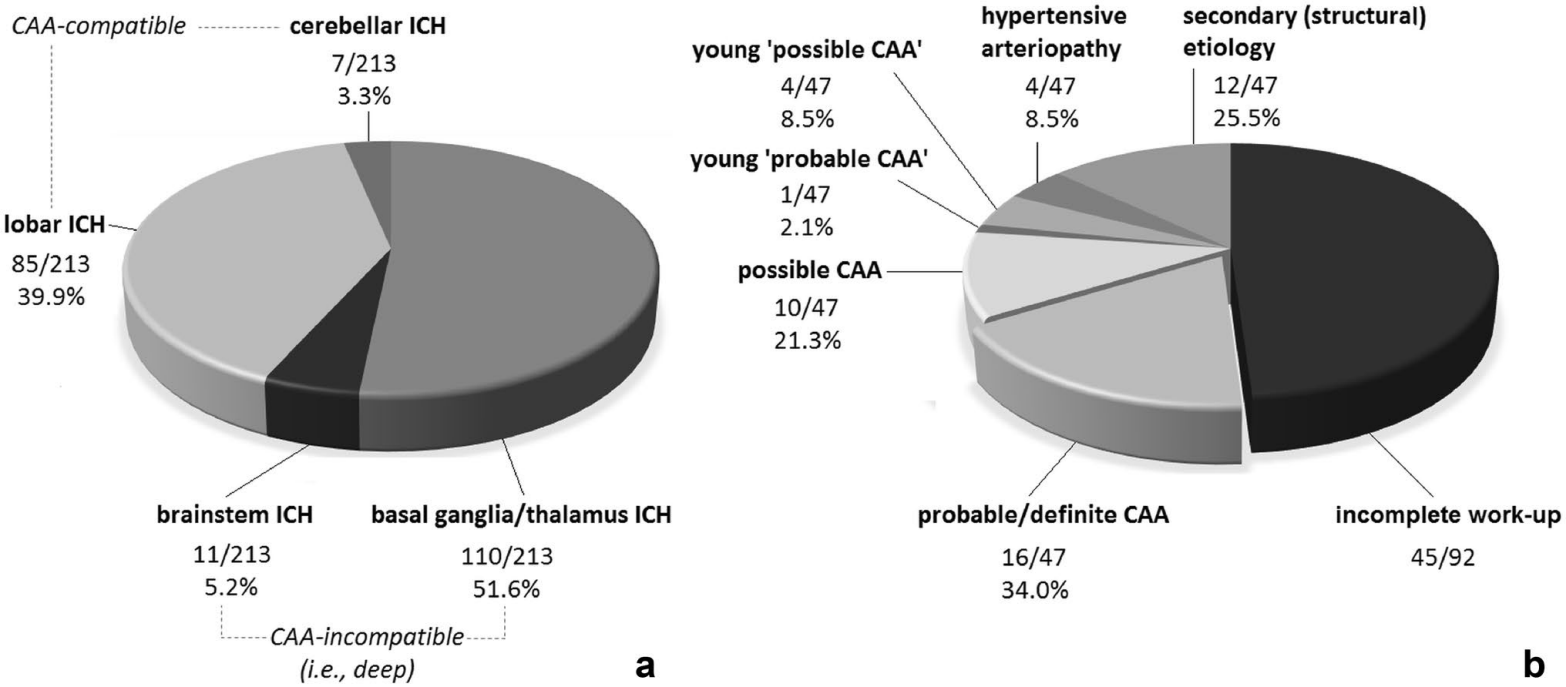
**a** Localization of spontaneous intracerebral hemorrhages (ICHs). **b** The distribution of underlying etiologies within lobar/cerebellar ICHs

A total of 324 patients having received any intracranial hemorrhage-related ICD codes as leading diagnosis in the given period were identified. After exclusions, 213 spontaneous ICHs were identified (Fig. 1). Among spontaneous ICHs, 121 deep ICHs [110 (51.6%) localized to the basal ganglia/thalamus and 11 (5.2%) to the brainstem) and 92 lobar/cerebellar ICHs (85 (39.9%) localized to any cerebral lobe and 7 (3.3%) to the cerebellum] were detected (Fig. 2a).

**Fig. 3:**
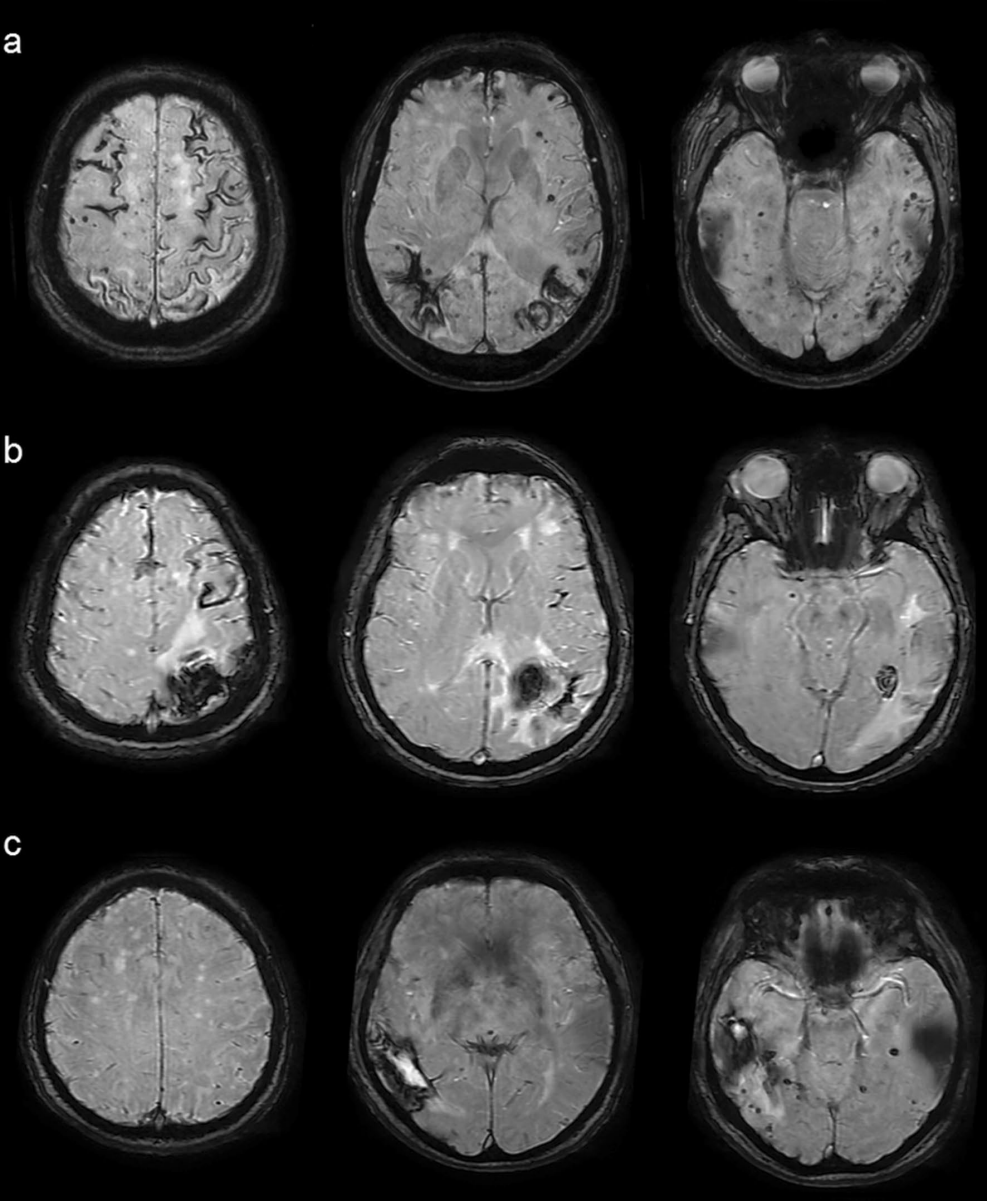
Representative axial MRI-SWI images of probable CAA patients at different parts of the spectrum. **a** Diffuse CSS with multiple lobar CMBs and ICHs of different ages. **b** Diffuse (but less extensive) CSS with a recurrent lobar ICH and a single CMB. **c** No CSS but multiple lobar CMBs accompanying a recent lobar ICH. The deep structures (i.e., basal ganglia, thalamus, and brainstem) are consistently devoid of hemorrhagic pathology. *CAA* cerebral amyloid angiopathy, *CMB* cerebral microbleed, *CSS* cortical superficial siderosis, *ICH* intracerebral hemorrhage, *MRI* magnetic resonance imaging, *SWI* susceptibility-weighted imaging

Out of lobar/cerebellar ICHs, 47 had ‘complete’ clinical work-up, of whom 2 proved to be definite CAA post mortem (Online Resource 1) and 14 were consistent with the diagnosis of probable CAA clinically (Fig. 3; one of them also became definite post mortem), rendering (16/47) 34.0% of all ‘completely’ worked-up lobar/cerebellar ICHs and (considering this rate as representative for all lobar/cerebellar ICHs) an estimated 14.7% of all spontaneous ICHs to be CAA-related. CMBs were present in 92.9% of probable CAA cases, with 42.9% having > 10 CMBs, whereas CSS was present in 78.6%, with 57.1% of probable CAA cases having diffuse CSS. In addition, ten patients met the criteria for possible CAA, and another five patients would have also met the criteria for probable (1) and possible CAA (4) except for their age being under 55. In 12 cases (25.5%), structural (i.e., secondary) etiologies such as arteriovenous malformation, ruptured aneurysm, sinus thrombosis, dural arteriovenous fistula or metastatic tumor were detected, whereas 4 cases (8.5%) were consistent with hypertensive arteriopathy (Fig. 2b). Out of the 14 probable CAA cases identified, originally only 4 had received CAA at least as suspected diagnosis (28.6%).

### Analysis of possible discriminators of ICH subgroups

The median age of the 213 patients with spontaneous ICH was 69.1 [60.3–79.0] years, with the lobar/cerebellar ICH group being significantly older compared to deep ICHs (74.5 [65.9–82.0] vs. 64.7 [57.9–76.6] years; *p* < 0.001; Table 1).

**Table 1:** Discriminators of spontaneous ICHs with regard to localization

	Lobar/cerebellar	Deep	MW/Chi^2^	multivariate logistic regression	
	ICH	ICH	*p*	*p*	OR (95% CI)
Patient number	92	121	–	–	
**Age at event**^**a**^**(y)**	**74.5 [65.9–82.0]**	**64.7 [57.9–76.6]**	** < 0.001**	**0.014**	**1.03 (1.01–1.06)**
**Sex (male/female) (%)**	**52.2**	**66.9**	**0.029**	> 0.05	–
Prior ischemic stroke (%)	12.0	12.7	> 0.05	–	–
Prior intracranial hemorrhage (%)	7.7	8.5	> 0.05	–	–
Prior TIA (TFNE) (%)	14.1	7.6	> 0.05	–	–
Prior loss of consciousness (%)	9.8	6.8	> 0.05	–	–
Family history for any stroke (%)	37.5	28.8	> 0.05	–	–
Anticoagulant use (%)	20.9	13.4	> 0.05	–	–
INR > 1.4 (%)	18.4	11.4	> 0.05	–	–
**Antiplatelet use**^**a**^**(%)**	**43.3**	**23.7**	**0.003**	**0.043**	**1.96 (1.02–3.75)**
**Combined antithrombotic use (%)**	**13.3**	**3.4**	**0.016**	> 0.05	–
**Hypertensive excess**^**a**^**(%)**	**48.9**	**71.2**	**0.001**	**0.002**	**0.39 (0.21–0.71)**
Chronic hypertension (%)	88.0	90.9	> 0.05	–	–
Case fatality (1-month) (%)	34.8	33.1	> 0.05	–	–

MW/Chi^2^, Mann–Whitney test (for Age at event) or Chi^2^ test (for other variables), *CI* confidence interval, *ICH* intracerebral hemorrhage, *INR* international normalized ratio, *OR* odds ratio, *TIA* transient ischemic attack, *TFNE* transient focal neurological episode, *y* year (median [interquartile range])

^a^Indicates significant predictors in the multivariate analyses

Bold font indicates variables with significant difference in univariate analyses

The distribution of sex was significantly different between deep and lobar/cerebellar ICH groups (*p* = 0.029), with a remarkable male preponderance in deep ICHs (66.9%) and a close to even ratio in lobar/cerebellar ICHs.

The frequency of prior episode(s) of TIA (TFNE), ischemic stroke, intracranial hemorrhage, and loss of consciousness in all ICHs were 10.5%, 12.4%, 8.1%, and 8.1%, respectively, being comparable between deep and lobar/cerebellar ICH groups.

The family history for either ischemic or hemorrhagic stroke (specification was not possible) was positive in 32.5%, with no between-group difference.

A total of 16.7% of ICH cases were on anticoagulant therapy at presentation, 74.3% because of AF. Three-quarter (77.1%) of anticoagulated patients were on a vitamin K antagonist [VKA; warfarin (4/27) or acenocoumarol (23/27)], 88.9% of whom had an INR > 1.4 at presentation. Two patients were on rivaroxaban, one on apixaban, together making up 8.6% of anticoagulated patients, while other direct oral anticoagulants (DOACs) were not represented. In five patients (14.3%), different doses of low-molecular-weight heparin (LMWH) were used. The frequency of anticoagulant use at presentation was comparable between ICHs in deep and lobar/cerebellar localizations. Notably, out of the seven lobar/cerebellar ICH patients with a positive history of intracranial hemorrhage, three were on therapeutic anticoagulation, and two of them were on antiplatelet treatment as well. Antiplatelet use was present in 32.2%, with a significant preponderance in lobar/cerebellar (43.3%) compared to deep ICHs (23.7%; *p* = 0.003). Altogether 7.7% of ICH patients were on combined antithrombotic regimen (on both anticoagulant and antiplatelet therapy), the significant majority (75.0%) suffering a lobar/cerebellar ICH (*p* = 0.016).

A total of 191 ICH patients were known for chronic hypertension (89.7%), which was the most prevalent risk factor for both deep and lobar/cerebellar ICHs, with no significant between-group difference. On the other hand, 61.5% of ICH patients experienced hypertensive excess (systolic blood pressure > 180 mmHg) at presentation, in a significantly higher rate in the deep compared to the lobar/cerebellar ICH group (71.2% vs. 48.9%, respectively, *p* = 0.001).

Analyzing the risk factors with significant between-group difference (age, sex, antiplatelet use, combined antithrombotic treatment, and hypertensive excess at presentation) in a multivariate binary logistic regression model revealed advanced age [*p* = 0.014; odds ratio (OR) = 1.03] and antiplatelet use (*p* = 0.043; OR = 1.96) to be statistically significant independent predictors of a lobar/cerebellar ICH, and hypertensive excess to be a strong significant independent predictor of a deep ICH (*p* = 0.002; OR = 0.39).

### Case fatality

The 1-month case fatality of ICH patients was 33.8%, with no significant difference between deep and lobar/cerebellar ICH groups (Table 1). Significant determinants of 1-month case fatality in ICHs as a whole were age, prior TIA, current anticoagulant use, and INR > 1.4 in univariate comparisons, with only advanced age (*p* = 0.003; OR = 1.04) and INR > 1.4 (*p* = 0.035; OR = 2.51) proven to be independent predictors of case fatality in multivariate analysis (Online Resource 2).

### Analysis of factors to predict CAA

The probable/definite CAA subgroup had the highest mean age at ICH presentation (75.9 ± 2.3 years), significantly higher compared to non-probable CAA patients (65.6 ± 1.1 years; *p* = 0.002). This was associated with a significant female predominance in probable/definite CAA (62.5%) as opposed to the male predominance (64.5%) in the comparator (*p* = 0.035).

Some 31.3% of probable/definite CAA cases had prior clinical event(s) of intracranial hemorrhage and exactly the same rate had prior TIA/TFNE, significantly higher than in the non-probable CAA group [6.8% (*p* = 0.008) and 7.4% (*p* = 0.010), respectively].

The ratio of patients on antiplatelet therapy (56.3%) within the definite/probable CAA subgroup was remarkably higher compared to non-probable CAA patients [25.2% (*p* = 0.009)].

Other factors and case fatality were not significantly different in the comparative analyses; of note, chronic hypertension was invariably prominent (Table 2).

**Table 2:** Discriminators of spontaneous ICHs with regard to probable/definite CAA diagnosis

	Probable/definite	Non-probable	St/Chi^2^	multivariate logistic regression	
	CAA	CAA	*p*	*p*	OR (95% CI*)*
Patient number	16	152	–	–	
**Age at event**^**a**^**(y)**	**75.9 ± 2.3**	**65.6 ± 1.1**	**0.002**	**0.012**	**1.08 (1.02–1.15)**
**Sex (male/female) (%)**	**37.5**	**64.5**	**0.035**	> 0.05	–
Prior ischemic stroke (%)	18.8	10.7	> 0.05	–	–
**Prior intracranial hemorrhage**^**a**^**(%)**	**31.3**	**6.8**	**0.008**	**0.005**	**8.53 (1.94–37.58)**
**Prior TIA (TFNE) (%)**	**31.3**	**7.4**	**0.010**	> 0.05	–
Prior loss of consciousness (%)	18.8	6.0	> 0.05	–	–
Family history for any stroke	42.9	29.1	> 0.05	–	–
Anticoagulant use (%)	18.8	12.8	> 0.05	–	–
INR > 1.4 (%)	20.0	10.4	> 0.05	–	–
**Antiplatelet use**^**a**^**(%)**	**56.3**	**25.2**	**0.009**	**0.042**	**3.45 (1.05–11.38)**
Combined antithrombotic use (%)	6.4	4.1	> 0.05	–	–
Hypertensive excess (%)	46.7	64.2	> 0.05	–	–
Chronic hypertension (%)	93.8	88.8	> 0.05	–	–
Case fatality (1-month) (%)	31.3	28.9	> 0.05	–	–

St/Chi^2^, Student *t* test (for Age at event) or Chi^2^ test (for other variables); *CI* confidence interval, *ICH* intracerebral hemorrhage, *INR* international normalized ratio, *OR* odds ratio, *TIA* transient ischemic attack, *TFNE* transient focal neurological episode, *y* year (mean ± SEM)

^a^Indicates significant predictors in multivariate analyses

Bold font: indicates variables with significant difference in univariate analyses

Multivariate analysis of factors significant in the univariate comparative analyses revealed older age (*p* = 0.012; OR = 1.08), prior intracranial hemorrhage (*p* = 0.005; OR = 8.53), and antiplatelet use (*p* = 0.042; OR = 3.45) as independent significant predictors of definite/probable CAA diagnosis.

## Discussion

Aiming to assess the predictors and outcome of spontaneous ICHs of different localization with particular focus on the prevalence of underlying CAA, this study identified hypertensive excess and younger age as independent predictors of deep whereas antiplatelet use of lobar/cerebellar localization, advanced age and INR > 1.4 as indepenent predictors of 1-month case fatality, and advanced age, prior intracranial hemorrhage, and antiplatelet use as independent predictors of probable/definite CAA diagnosis, in addition to an estimated prevelance of CAA-related ICHs comparable to prior publications.

The timely identification of patients with probable CAA is imperative, as it is associated with serious therapeutic consequences, especially regarding the avoidance of antithrombotic/thrombolytic medications, with increasing literature demonstrating a higher risk of harm compared to benefit (Yamada 2015). Despite these, CAA is considered to be underdiagnosed worldwide, its epidemiology is largely based on neuropathological case series, and the prevalence of CAA among ICH patients (i.e., CAA-related ICH) with/without associated potential risk factors have only been addressed by a few studies on clinical grounds (Online Resource 3) (Meretoja et al. 2012; Yeh et al. 2014; Roh et al. 2018; Jamieson et al. 2012; Palm et al. 2013). Consequently, our aim was to revise all spontaneous ICH cases in our center in a 4-year period, with special focus on identifying patients with probable/definite CAA, and analyzing associated risk factors and fatal outcome.

The 213 ICH cases detected represent an incidence of approximately 13.3/100,000 persons/year, resembling the 12–15/100,000 persons/year reported in the U.S. (Aguilar and Brott 2011). In our study, 51.6% of spontaneous ICHs originated in the basal ganglia/thalamus, 3.3% in the cerebellum, 5.2% in the brainstem, and 39.9% were of lobar localization. According to international data reporting 35–70%, 5–10%, 5–10%, and 15–30%, respectively (Aguilar and Brott 2011), this suggests a relative overrepresentation of lobar ICHs compared to expectations, highlighting the relevance and necessity of an increased awareness of CAA in this population. This ratio is similar to Swedish and US findings with 43.2% and 40.5% rates of lobar ICHs, respectively (Zia et al. 2007; Broderick et al. 1993). The analysis of risk factors confirmed ICH as the disease of the elderly [the median 69.1 years being consistent with previous reports (O'Donnell et al. 2010)]. In particular, older age proved to be an independent predictor of lobar/cerebellar (i.e., CAA-compatible) ICH localization, resembling findings for lobar ICHs (Labovitz et al. 2005; Matsukawa et al. 2012; Weimar et al. 2011). Hypertension was by far the most common coexistent factor (~ 90%) irrespective of ICH localization. Identifying hypertension as primary risk factor for ICHs is consistent with international meta-analyses (Ikram et al. 2012; O'Donnell et al. 2010; Ariesen et al. 2003); however, its prevalence was higher than in many individual studies (Zia et al. 2007; Yeh et al. 2014; Broderick et al. 1993), albeit similar to some reports from Europe (Smajlovic et al. 2008; Safatli et al. 2016). Despite the concept that chronic hypertension would associate more with deep ICHs, our results emphasize that it is essentially present in any subtypes of ICHs [in line with some prior observations (Broderick et al. 1993)] and only an extreme hypertension around the event demonstrated to be a significant (in fact the strongest) predictor of deep ICHs. Male sex, a factor frequently reported as a risk for ICH (Labovitz et al. 2005; Ariesen et al. 2003; van Asch et al. 2010), was also slightly overrepresented in the pooled cohort (60.6%), driven, however, entirely by deep ICHs (66.9%), with the sex rate of lobar/cerebellar ICHs being ~ 50%, recapitulating prior observations (Labovitz et al. 2005; Zia et al. 2007; Yeh et al. 2014). The use of antithrombotics were frequent (41.1%), with antiplatelet use proven to be an independent predictor of lobar/cerebellar ICH localization. The results were practically identical when using the traditional lobar vs. non-lobar comparison (Online Resource 4).

The 1-month case fatality of all ICHs was 33.8%, with no significant effect of localization. This rate is consistent with a previous report from this region (Ovary et al. 2004) and similar to reports from the US (Labovitz et al. 2005), being somewhat favorable compared to international median of 40.4% in a recent meta-analysis (van Asch et al. 2010). The identification of age as an independent decisive factor recapitulates this meta-study (van Asch et al. 2010). Though resembling findings of SMASH-U studies for medication-related ICHs (Meretoja et al. 2012; Yeh et al. 2014), the independent prognostic value of admission INR > 1.4 (but not of anticoagulant use per se in the multivariate analysis) is a novelty [to our knowledge reported previously only in primary lobar ICHs (Falcone et al. 2013)], giving an additional context to the risk posed by anticoagulants, particularly VKAs.

The analysis of potential clinical predictors of CAA-related ICHs revealed older age (~ 8% increase in risk per year) and prior intracranial hemorrhage (~ 8.5-fold risk) as independent significant predictors of probable/definite CAA diagnosis, which is consistent with the notion that CAA is the disease of the elderly (Yamada 2015) and CAA-related ICHs are often recurrent (Biffi and Greenberg 2011). These, together with the female predominance in probable/definite CAA (though proven to be not independent of age) recapitulate key observations of a recent study from the US comparing their probable/definite CAA-related ICH group with hypertension-related ICHs (as per SMASH-U) (Roh et al. 2018), and concord with autopsy studies demonstrating female predominance in CAA-related ICHs (Attems et al. 2008; Hirohata et al. 2010).

Highlighting its primary role in ICH development irrespective of etiology, our data indicates that pre-existing hypertension is invariably associated with definite/probable CAA diagnosis (93.8%), with the prevalence of hypertensive excess at presentation (46.7%) not being significantly different from the comparator group either.

Though significant only at the univariate level, a remarkably high rate of definite/probable CAA patients (31.3%) had experienced prior ‘TIA’ compared to non-probable CAA (7.4%), presumably representing ‘amyloid spells’, suggested to be of epileptic origin. Interpreting these events as ‘ischemic’ necessarily adds to the inherited risk of ICH in CAA, due to the consequent initiation of antiplatelet therapy. Indeed, antiplatelet and anticoagulant drugs are considered a risk for ICH in CAA. Our study concords with this, revealing 68.8% of ICH patients with definite/probable CAA to be under at least one type of antithrombotic medication, with the multivariate analysis identifying antiplatelet (but not anticoagulant) use as an independent predictor of probable/definite CAA diagnosis. The 3.3-times higher prevalence of probable/definite CAA diagnosis in antiplatelet users vs. non-users resembles the twofold prevalence of lobar microbleeds in antiplatelet user ICH patients in a previous study (Gregoire et al. 2010).

Underlying the relevance of missed diagnoses, a patient with fatal CAA-related ICH was on combined antithrombotic therapy despite a prior lobar ICH 1 year before the index ICH, having an SWI scan at first ICH already consistent with probable CAA, retrospectively.

Our study has a number of limitations, including its retrospective nature, resulting in a certain amount of random missing values regarding some clinical factors analyzed, decreasing their statistical power. Strengths include the relatively large subject number, to our knowledge being among the largest studies reporting multivariate binary analysis of clinical discriminators of ICH localization, overtaken by a study from Japan (Matsukawa et al. 2012) and Germany (Weimar et al. 2011). Additional strength is the unprecedented rigor that probable and possible CAA diagnoses were established in (and thus CAA-related ICH prevalence estimated based on) a subgroup with ‘complete’ work-up including MRI-SWI and angiography (not allowing CT-only), increasing diagnostic sensitivity and specificity.

## Conclusion

This study provides an in-depth retrospective analysis of spontaneous ICHs, with particular focus on the prevalence and clinical predictors of CAA-related ICH, first in a country from this region, and among the few reports previously published in the literature. We conclude that CAA-related ICHs are at least as frequent (14.7%) in our population as reported internationally (5–20%) (Biffi and Greenberg 2011). Notably, the remarkably low rate of clinically and radiologically established probable CAA diagnosis puts a significant percentage of the population, especially the elderly under antiplatelet therapy (as demonstrated), at a high risk of possibly lethal ICHs. This highlights the need for an increased awareness of CAA by both neurologists and radiologists.

## Electronic supplementary material

Below is the link to the electronic supplementary material.

Supplementary file1 (PDF 523 kb)

Supplementary file2 (PDF 150 kb)

Supplementary file3 (PDF 151 kb)

Supplementary file4 (PDF 154 kb)
