# Correction to: Predictors of localization, outcome, and etiology of spontaneous intracerebral hemorrhages: focus on cerebral amyloid angiopathy

**DOI:** 10.1007/s00702-020-02195-x

**Published:** 2020-05-09

**Authors:** Bernadett Fakan, Zita Reisz, Denes Zadori, Laszlo Vecsei, Peter Klivenyi, Levente Szalardy

**Affiliations:** 1 Department of Neurology, Faculty of Medicine, Albert Szent-Györgyi Clinical Center, University of Szeged, Semmelweis u. 6, 6725 Szeged, Hungary; 2 Department of Pathology, Faculty of Medicine, Albert Szent-Györgyi Clinical Center, University of Szeged, Állomás u. 2, 6725 Szeged, Hungary; 3 MTA-SZTE Neuroscience Research Group, Semmelweis u. 6, 6725 Szeged, Hungary

## Correction to: Journal of Neural Transmission 10.1007/s00702-020-02174-2

The original version of this article unfortunately contained a mistake. Mistake in Tables 1 and 2.

Indeed, the Tables 1 and 2 has a superscript "a" as footnote of variables with significant difference in the multivariate model. This superscript "a" originally was before the measure in the bracket (i.e. (y) or (%)), as approved by the authors in the proof. However, the superscript "a" was not before but after the measure in the bracket yielding e.g. (y)a, in the first published version of the manuscript in Tables 1 and 2.

However, this change in fact resulted in a typo in Table 1, namely "Age at eventa" with an attached "a" after "event" in the same font size.

The corrected Tables 1 and 2 are placed in the following page.

The original article has been corrected.

**Table 1:** Discriminators of spontaneous ICHs with regard to localization

	Lobar/cerebellar	Deep	MW/Chi^2^	multivariate logistic regression	
	ICH	ICH	*p*	*p*	OR (95% CI)
Patient number	92	121	–	–	
**Aage at event**^a^** (y)**	**74.5 [65.9–82.0]**	**64.7 [57.9–76.6]**	** < 0.001**	**0.014**	**1.03 (1.01–1.06)**
**Sex (male/female) (%)**	**52.2**	**66.9**	**0.029**	> 0.05	–
Prior ischemic stroke (%)	12.0	12.7	> 0.05	–	–
Prior intracranial hemorrhage (%)	7.7	8.5	> 0.05	–	–
Prior TIA (TFNE) (%)	14.1	7.6	> 0.05	–	–
Prior loss of consciousness (%)	9.8	6.8	> 0.05	–	–
Family history for any stroke (%)	37.5	28.8	> 0.05	–	–
Anticoagulant use (%)	20.9	13.4	> 0.05	–	–
INR > 1.4 (%)	18.4	11.4	> 0.05	–	–
**Antiplatelet use**^a^** (%)**	**43.3**	**23.7**	**0.003**	**0.043**	**1.96 (1.02–3.75)**
**Combined antithrombotic use** (%)	**13.3**	**3.4**	**0.016**	> 0.05	–
**Hypertensive excess**^a^** (%)**	**48.9**	**71.2**	**0.001**	**0.002**	**0.39 (0.21–0.71)**
Chronic hypertension (%)	88.0	90.9	> 0.05	–	–
Case fatality (1-month) (%)	34.8	33.1	> 0.05	–	–

MW/Chi^2^, Mann–Whitney test (for Age at event) or Chi^2^ test (for other variables), *CI* confidence interval, *ICH* intracerebral hemorrhage, *INR* international normalized ratio, *OR* odds ratio, *TIA* transient ischemic attack, *TFNE* transient focal neurological episode, *y* year (median [interquartile range])

^a^Indicates significant predictors in the multivariate analyses

Bold font indicates variables with significant difference in univariate analyses

**Table 2:** Discriminators of spontaneous ICHs with regard to probable/definite CAA diagnosis

	Probable/definite	Non-probable	St/Chi^2^	multivariate logistic regression	
	CAA	CAA	*p*	*p*	OR (95% CI*)*
Patient number	16	152	–	–	
**Age at event**^a^** (y)**	**75.9 ± 2.3**	**65.6 ± 1.1**	**0.002**	**0.012**	**1.08 (1.02–1.15)**
**Sex (male/female) (%)**	**37.5**	**64.5**	**0.035**	> 0.05	–
Prior ischemic stroke (%)	18.8	10.7	> 0.05	–	–
**Prior intracranial hemorrhage**^a^** (%)**	**31.3**	**6.8**	**0.008**	**0.005**	**8.53 (1.94–37.58)**
**Prior TIA (TFNE) (%)**	**31.3**	**7.4**	**0.010**	> 0.05	–
Prior loss of consciousness (%)	18.8	6.0	> 0.05	–	–
Family history for any stroke	42.9	29.1	> 0.05	–	–
Anticoagulant use (%)	18.8	12.8	> 0.05	–	–
INR > 1.4 (%)	20.0	10.4	> 0.05	–	–
**Antiplatelet use**^a^** (%)**	**56.3**	**25.2**	**0.009**	**0.042**	**3.45 (1.05–11.38)**
Combined antithrombotic use (%)	6.4	4.1	> 0.05	–	–
Hypertensive excess (%)	46.7	64.2	> 0.05	–	–
Chronic hypertension (%)	93.8	88.8	> 0.05	–	–
Case fatality (1-month) (%)	31.3	28.9	> 0.05	–	–

St/Chi^2^, Student *t* test (for Age at event) or Chi^2^ test (for other variables); *CI* confidence interval, *ICH* intracerebral hemorrhage, *INR* international normalized ratio, *OR* odds ratio, *TIA* transient ischemic attack, *TFNE* transient focal neurological episode, *y* year (mean ± SEM)

^a^Indicates significant predictors in multivariate analyses

Bold font: indicates variables with significant difference in univariate analyses

