# Supplementary material for: Predictors of localization, outcome, and etiology of spontaneous intracerebral hemorrhages: focus on cerebral amyloid angiopathy
Source: J Neural Transm (Vienna). 2020 Mar 19;127(6):963–72. doi: 10.1007/s00702-020-02174-2 (PMC7248013; doi:10.1007/s00702-020-02174-2)
Supplement: Supplementary file 1 — Supplementary file1 (PDF 523 kb) [file 702_2020_2174_MOESM1_ESM.pdf]

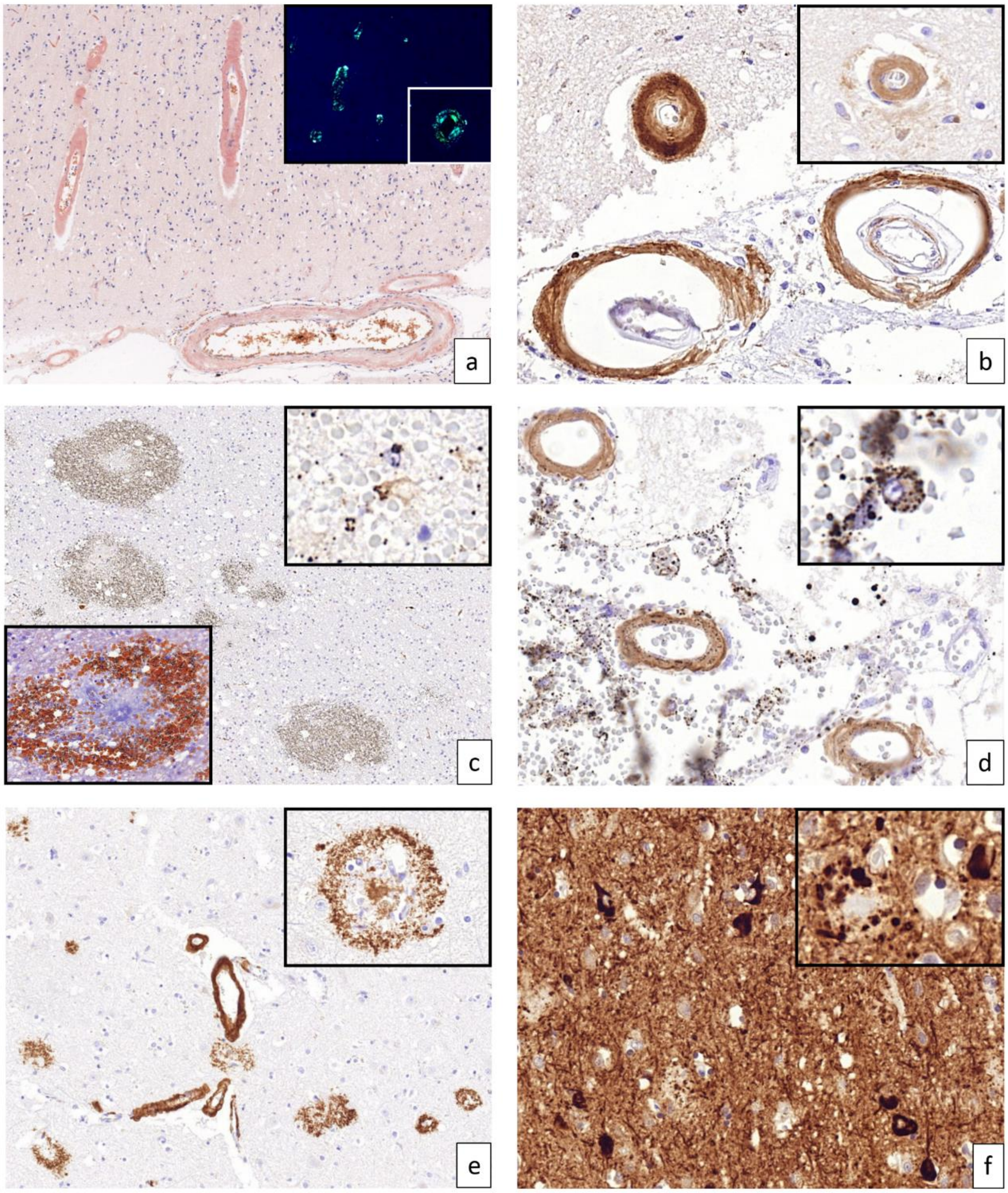

Online Resource 1 Neuropathology of definite cerebral amyloid angiopathy (CAA) cases. a) Leptomeningeal and penetrating cortical arteries with thickened Congo red-positive walls, demonstrating apple-green birefringence under the polarized microscope (insets). b) Immunostaining for amyloid-β reveals denegerated vessel walls with typical double-barrel formation, with occasional widening of the perivascular space and perivascular amyloid-β deposition (inset). c) Subcortical microbleeds with erythrocyte extravasation, hemosiderin deposition, and characteristic central pallor with a central arteriole/capillary (often occluded; lower-left inset (Crossmon)), occasionally with immunopositivity for amyloid-β (upper-right inset). d) Cortical superficial (i.e., subarachnoid) siderosis with apparent siderophage activity (occasionally also laden with diffuse amyloid-β-immunopositive material; inset). e) All 3 patients with definite CAA demonstrated different amount of amyloid-β plaque pathology in addition to CAA. f) Only 2 of the 3 definite CAA patients showed Tau pathology in the examined regions with neurofibrillary tangles and dystrophic neurites (inset), reflecting the ~70% overlap with Alzheimer's disease pathology.
