# Supplementary material for: Predictors of localization, outcome, and etiology of spontaneous intracerebral hemorrhages: focus on cerebral amyloid angiopathy
Source: J Neural Transm (Vienna). 2020 Mar 19;127(6):963–72. doi: 10.1007/s00702-020-02174-2 (PMC7248013; doi:10.1007/s00702-020-02174-2)
Supplement: Supplementary file 2 — Supplementary file2 (PDF 150 kb) [file 702_2020_2174_MOESM2_ESM.pdf]

Online Resource 2 Predictors of 1-month case fatality in spontaneous ICHs

		**Case fatality at 1 month**	**Alive at 1 month**	**MW/Chi^2**	**multivariate logistic regression**	
				p	***p***	***OR* (95% CI)**
Patient number		72	138	-	-	-
**Age at event***	**y**	**75.3 [66.4–82.4]**	**65.4 [56.3–76.8]**	**<0.001**	**0.003**	**1.04 (1.01–1.07)**
Sex (male/all)	%	65.3	58.0	>0.05	-	-
Localization (deep/all)	%	55.6	58.7	>0.05	-	-
Prior ischemic stroke	%	15.9	10.9	>0.05	-	-
Prior intracranial hemorrhage	%	7.2	8.8	>0.05	-	-
**Anticoagulant use**	**%**	**25.7**	**11.7**	**0.010**	>0.05	-
**INR>1.4***	**%**	**23.6**	**8.7**	**0.004**	**0.035**	**2.51 (1.07–5.88)**
Antiplatelet use	%	29.0	33.8	>0.05	-	-
Combined antithrombotic use	%	10.1	5.9	>0.05	-	-
Hypertensive excess	%	66.7	60.2	>0.05	-	-
Chronic hypertension	%	88.9	89.9	>0.05	-	-

MW/Chi^2, data obtained from the Mann-Whitney test (for Age at event) or the Chi square test (for all other variables); CI, confidence interval; ICH, intracerebral hemorrhage; INR, international normalized ratio; *OR*, odds ratio; TIA, transient ischemic attack; TFNE, transient focal neurological episode; y, year (data is presented in median [interquartile range]); bold font indicates variables with significant difference in comparative analyses; * indicates significant predictors in the model controlling for all variables significant in the comparative analyses. Three cases were excluded from the analyses due to unrelated cause of death, as per definition.
