# Supplementary material for: Predictors of localization, outcome, and etiology of spontaneous intracerebral hemorrhages: focus on cerebral amyloid angiopathy
Source: J Neural Transm (Vienna). 2020 Mar 19;127(6):963–72. doi: 10.1007/s00702-020-02174-2 (PMC7248013; doi:10.1007/s00702-020-02174-2)
Supplement: Supplementary file 3 — Supplementary file3 (PDF 151 kb) [file 702_2020_2174_MOESM3_ESM.pdf]

Online Resource 3 Overview of studies reporting CAA-related ICH prevalence on clinical grounds. The studies are separated based on whether they reported subgroups of CAA diagnosis or not.

	**Jamieson 2012** [1][a]		**Meretoja 2012** [2] SMASH-U		**Palm 2013** [3] semi-SMASH-U[a]		**Roh 2018** [4] semi-SMASH-U[a]		**Present study**	
definite CAA	0	0.0%	10	4.9%	0	0.0%	2	2.0%	3	11.5%
supportive histopathology for CAA	0	0.0%	2	1.0%	0	0.0%	6	6.0%	0	0.0%
probable CAA	6	11.3%	36	17.6%	15	34.1%	47	46.5%	13	50.0%
possible CAA	47	88.7%	157	76.6%	29	65.9%	46	45.5%	10	38.5%
all ICHs (spontaneous)	136		1013		152		425		213	
all CAAs / all ICHs	53	39.0%	205	20.2%	44	28.9%	101	23.8%	26/47/92	23.9%[c]
probable-definite CAAs / all ICHs	6	4.4%	48	4.7%	15	9.9%	55	12.9%	16/47/92	14.7%[c]
MRI / all ICHs	NA		15.1%		20.4%		NA		37.1%	
MRI / all CAAs	NA		20.5%		NA		71.3%[b]		96.2%[d]	
SWI / all ICHs	NA		NA		NA		NA		31.0%	
SWI / CAAs	NA		NA		NA		NA		92.3%[d]	
angiography / all ICHs	NA		25%		26.3%		NA		55.4%	
angiography / all CAAs	NA		NA		NA		NA		100.0%	
cerebellar ICHs excluded from CAA	yes		yes		yes		no		no	

	**Yeh 2014** [5] SMASH-U		**Cappellari 2015** [6] semi-SMASH-U[e]		**Owolabi 2017** [7] SMASH-U		**Wu 2017** [8] SMASH-U		**Forlivesi 2018** [9] SMASH-U	
definite CAA	NA		NA		NA		NA		NA	
supportive histopathology for CAA	NA		NA		NA		NA		NA	
probable CAA	NA		NA		NA		NA		NA	
possible CAA	NA		NA		NA		NA		NA	
all CAAs / all ICHs	463	12.2%	62	14.6%	1	2.4%	344	23.7%	51	16.6%
all ICHs (spontaneous)	3785		424		42		1452		308	
probable-definite CAA / all ICHs	NA		NA		NA		NA		NA	
MRI / all ICHs	NA		NA		NA		NA		NA	
MRI / all CAAs	NA		NA		NA		NA		NA	
SWI / all ICHs	NA		NA		NA		NA		NA	
SWI / CAAs	NA		NA		NA		NA		NA	
angiography / all ICHs	25.0%		NA		NA		NA		NA	
angiography / all CAAs	39.1%		NA		NA		NA		NA	
cerebellar ICHs excluded from CAA	yes		no		yes		yes		yes	

CAA, cerebral amyloid angiopathy, ICH, intracerebral hemorrhage; MRI, magnetic resonance imaging; NA, not available; SWI, susceptibility-weighted imaging;

[a] structural etiologies not included (i.e., primary ICHs only)

[b] all probable/supported/definite CAA but 3 had MRI (SWI rate undisclosed); those without MRI had supportive intraoperative histology.

[c] data extrapolated from the rate within adequately worked-up lobar/cerebellar ICHs (i.e., MRI-SWI and angiography or neuropathology required), as detailed in the manuscript.

[d] all probable/definite CAA but 1 had MRI and all but 2 had SWI; those without MRI/SWI had definite CAA diagnosis post mortem.

[e] brainstem ICH was allowed in CAA.

References

1. Jamieson EI, Newman D, Metcalf AK, et al. (2012) Dementia is strongly associated with 90-day mortality in lobar cerebral amyloid angiopathy related intra-cerebral haemorrhage. J Neurol Sci 322 (1-2):161-165. http://doi.org/10.1016/j.jns.2012.07.047
2. Meretoja A, Strbian D, Putaala J, et al. (2012) SMASH-U: a proposal for etiologic classification of intracerebral hemorrhage. Stroke 43 (10):2592-2597. http://doi.org/ 10.1161/STROKEAHA.112.661603
3. Palm F, Henschke N, Wolf J, et al. (2013) Intracerebral haemorrhage in a population-based stroke registry (LuSSt): incidence, aetiology, functional outcome and mortality. J Neurol 260 (10):2541-2550. http://doi.org/10.1007/s00415-013-7013-0
4. Roh D, Sun CH, Schmidt JM, et al. (2018) Primary Intracerebral Hemorrhage: A Closer Look at Hypertension and Cerebral Amyloid Angiopathy. Neurocrit Care 29 (1):77-83. http://doi.org/10.1007/s12028-018-0514-z
5. Yeh SJ, Tang SC, Tsai LK, Jeng JS (2014) Pathogenetical subtypes of recurrent intracerebral hemorrhage: designations by SMASH-U classification system. Stroke 45 (9):2636-2642. http://doi.org/10.1161/STROKEAHA.114.005598
6. Cappellari M, Zivelonghi C, Moretto G, et al. (2015) The etiologic subtype of intracerebral hemorrhage may influence the risk of significant hematoma expansion. J Neurol Sci 359 (1-2):293-297. http://doi.org/10.1016/j.jns.2015.11.024
7. Owolabi M, Ogbole G, Akinyemi R, et al. (2017) Development and Reliability of a User-Friendly Multicenter Phenotyping Application for Hemorrhagic and Ischemic Stroke. J Stroke Cerebrovasc Dis 26 (11):2662-2670. http://doi.org/10.1016/j.jstrokecerebrovasdis.2017.06.042
8. Wu TY, Yassi N, Shah DG, et al. (2017) Simultaneous Multiple Intracerebral Hemorrhages (SMICH). Stroke 48 (3):581-586. http://doi.org/10.1161/STROKEAHA.116.015186
9. Forlivesi S, Turcato G, Zivelonghi C, et al. (2018) Association of Short- and Medium-Term Particulate Matter Exposure with Risk of Mortality after Spontaneous Intracerebral Hemorrhage. J Stroke Cerebrovasc Dis 27 (9):2519-2523. http://doi.org/10.1016/j.jstrokecerebrovasdis.2018.05.007
