# Supplementary material for: Predictors of localization, outcome, and etiology of spontaneous intracerebral hemorrhages: focus on cerebral amyloid angiopathy
Source: J Neural Transm (Vienna). 2020 Mar 19;127(6):963–72. doi: 10.1007/s00702-020-02174-2 (PMC7248013; doi:10.1007/s00702-020-02174-2)
Supplement: Supplementary file 4 — Supplementary file4 (PDF 154 kb) [file 702_2020_2174_MOESM4_ESM.pdf]

Online Resource 4 Discriminators of spontaneous ICHs with regard to lobar vs. non-lobar localization

				MW/Chi²	multivariate logistic regression	
		**Lobar ICH**	**Non-lobar ICH**	***p***	***p***	***OR* (95% CI)**
Patient number		85	128	-	-	
**Age at event***	**y**	**74.7 [66.2–82.2]**	**64.9 [57.8–76.6]**	**<0.001**	**0.006**	**1.04 (1.01–1.06)**
Sex (male/female)	%	52.9	65.6	>0.05	-	-
Prior ischemic stroke	%	12.9	12.0	>0.05	-	-
Prior intracranial hemorrhage	%	8.3	8.0	>0.05	-	-
Prior TIA (TFNE)	%	15.3	7.2	>0.05	-	-
Prior loss of consciousness	%	10.6	6.4	>0.05	-	-
Family history for any stroke	%	37.8	29.0	>0.05	-	-
Anticoagulant use	%	20.2	14.3	>0.05	-	-
INR>1.4	%	17.3	12.5	>0.05	-	-
**Antiplatelet use***	**%**	**44.6**	**24.0**	**0.002**	**0.041**	**1.97 (10.3–3.76)**
**Combined antithrombotic use**	**%**	**13.3**	**4.0**	**0.018**	>0.05	**-**
**Hypertensive excess***	**%**	**48.2**	**70.4**	**0.001**	**0.003**	**0.39 (0.21–0.72)**
Chronic hypertension	%	88.2	90.6	>0.05	-	-
Case fatality (1-month)	%	35.3	32.8	>0.05	-	-

MW/Chi², Mann-Whitney test (for Age at event) or Chi² test (for other variables); CI, confidence interval; ICH, intracerebral hemorrhage; INR, international normalized ratio; *OR*, odds ratio; TIA, transient ischemic attack; TFNE, transient focal neurological episode; y, year (median [interquartile range]); bold font indicates variables with significant difference in univariate analyses; * indicates significant predictors in the multivariate analyses.
